# Association of atrial fibrillation burden and clinical profile with blood biomarkers: Results from the ISOLATION Ablation Cohort

**DOI:** 10.1016/j.hroo.2025.02.017

**Published:** 2025-02-28

**Authors:** Zarina Habibi, Dominique V.M. Verhaert, Konstanze Betz, Ben J.M. Hermans, Joris Winters, Suzanne A.M. Philippens, Sevasti-Maria Chaldoupi, Bart Maesen, Jos G. Maessen, Aaron Isaacs, Sjoerd W. Westra, Robin Nijveldt, Ludovic Gillet, Ursula-Henrike Wienhues-Thelen, Merlin Koehler, Stef Zeemering, Kevin Vernooy, Dominik Linz, Ulrich Schotten

**Affiliations:** 1 Department of Physiology, Maastricht University, Maastricht, The Netherlands; 2 Cardiovascular Research Institute Maastricht (CARIM), Maastricht University, Maastricht, The Netherlands; 3 Department of Cardiology, Radboudumc, Nijmegen, The Netherlands; 4 Department of Cardiology, Maastricht UMC+, Maastricht, The Netherlands; 5 Eifelklinik St. Brigida GmbH & CO KG, Simmerath, Germany; 6 Department of Cardiothoracic Surgery, Maastricht UMC+, Maastricht, The Netherlands; 7 Maastricht Centre for Systems Biology (MaCSBio), Maastricht, The Netherlands; 8 Roche Diagnostics International Ltd. Rotkreuz Switzerland; 9 Roche Diagnostics GmbH Penzberg Germany

**Keywords:** Biomarkers, Atrial fibrillation, Catheter ablation, Preprocedural atrial fibrillation, Burden, Sex differences, BMP10

## Abstract

**Background:**

Advances have been made in identifying biomarkers for atrial fibrillation (AF) outcomes.

**Objective:**

The link between clinical determinants, especially AF burden, and blood biomarkers remains underexplored.

**Methods:**

We conducted a cross-sectional analysis of AF patients scheduled for catheter ablation in the ISOLATION study (July 2020–May 2022, NCT04342312). Patient characteristics and blood samples were collected before ablation. AF burden was assessed using hand-held electrocardiograms (ECGs) over 4 weeks. Blood samples were analyzed for biomarkers, including bone morphogenetic protein 10 (BMP10), angiopoietin-2 (Ang-2), fibroblast growth factor 23 (FGF23), and others. We trained elastic net regression models to identify the most important clinical determinants out of 64 available clinical features.

**Results:**

We analyzed blood samples from 508 patients with a mean age of 63 ±9 years; 31.1% were female. Of these, 70% had paroxysmal AF and 30% persistent AF. Heart failure was present in 15% of patients. In 140 patients (28%), AF was observed during blood draw. AF burden before ablation was available in 389 patients. After multivariable analysis, the following clinical determinants were independently associated with biomarker levels: AF burden, AF during blood draw, age, heart failure, decreased kidney function, and female sex. Most notably, AF burden and AF rhythm at the time of sampling were strongly associated with various biomarker levels. Female sex was positively associated with BMP10 and FGF23, but negatively associated with high sensitive Troponin-T (hs-TNT).

**Conclusions:**

AF burden is a strong determinant of many biomarkers, underpinning their relevance as covariates in biomarker studies. Pro-fibrotic biomarkers are increased in female patients, whereas male patients more often show elevated biomarkers of myocardial injury.

## Graphical abstract

**Figure:**
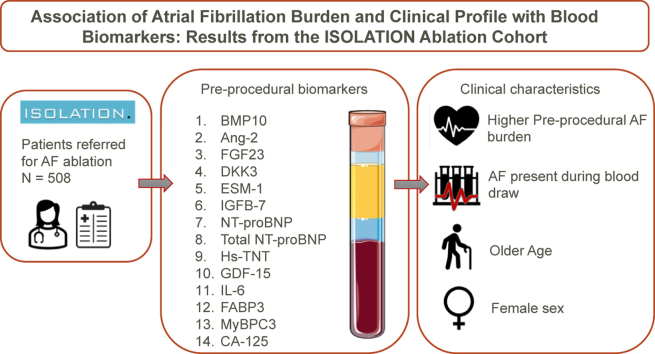

**Key findings**

▪ We identified preprocedural atrial fibrillation (AF) burden as a significant determinant of biomarker levels in patients undergoing catheter ablation.
▪ We highlighted the importance of rhythm at the time of blood sampling as a key factor influencing biomarker levels.
▪ Profibrotic biomarker levels such as BMP10 and FGF23 were higher in female patients, and biomarkers levels indicative for myocardial injury, such as hsTNT were higher in male patients.

## Introduction

Atrial fibrillation (AF) is the most frequent arrhythmia in adults and is associated with worsening of heart failure and an increased risk of stroke.^1^^,^^2^ Recently, significant advancements have been made in identifying blood biomarkers associated with AF. Many of these recently identified biomarkers carry prognostic value in patients with AF. The benchmark biomarker N-terminal pro-brain natriuretic peptide (NT-proBNP) has been shown to be associated with prevalence of AF, AF progression, and AF recurrences in patients undergoing AF ablation.^3^^,^^4^ Bone morphogenetic protein 10 (BMP10), a more recently discovered biomarker, has been shown to be associated with AF, AF recurrences, and stroke.^3^^,^^5^, ^6^, ^7^

A recent study in unselected patients presenting to the hospital has shown that fibroblast growth factor 23 (FGF23), BMP10, and angiopoietin 2 (Ang-2) were associated with AF prevalence.^3^ In a previous study, common cardiovascular biomarkers were analyzed in patients referred to the hospital with known cardiovascular risk factors, including AF. FGF23 was found as a biomarker to identify patients with AF.^8^ Furthermore, it was recently shown that the biomarkers NT-proBNP, Ang-2, and BMP10 could predict sinus rhythm at 12-month follow-up in patients diagnosed with AF.^9^ In addition to their prognostic value, biomarkers represent diverse pathophysiological pathways in AF, including inflammation, myocyte injury, collagen deposition, fatty infiltration,^10^^,^^11^ and structural remodeling.^11^, ^12^, ^13^

The individual pathophysiological mechanisms underlying AF vary from patient to patient, with some being more common than others. Notably, fibrosis stands out as 1 of the hallmarks of structural remodeling in patients with AF. Biomarkers such as BMP10 and FGF23 have been proposed as potential indicators of atrial fibrosis.^6^^,^^14^

Thus, biomarkers may provide information about individual disease mechanisms of AF, which potentially may impact the course of the arrhythmia. However, a notable gap exists in our understanding regarding the association between AF-associated blood biomarkers and clinical determinants, particularly AF burden. Recent studies show that AF burden might be important in the risk stratification of AF patients. A higher AF burden is associated with an increased risk of ischemic stroke in patients with paroxysmal AF. This knowledge may impact anticoagulation therapy, for example.^15^ Despite enormous progress in developing tools for rhythm monitoring, measuring AF burden remains a logistic challenge. More knowledge about the relation between AF burden and biomarkers is therefore desirable.

We present data from the prospective multicenter ISOLATION cohort study of patients undergoing AF catheter ablation, encompassing clinical characteristics, routine tests, and additional study procedures. Our aim is to explore the relationship between clinical determinants, preprocedural AF burden, and biomarker levels, thereby providing valuable context to these biomarkers in a population of AF patients scheduled for catheter ablation.

## Methods

### Study population

We performed a cross-sectional analysis of patients included in the prospective ISOLATION study (NCT04342312), a multicenter cohort study of AF patients scheduled for catheter ablation. Patients were consecutively enrolled from July 2020 through May 2022. The study protocol has been published.^16^ The research reported in this paper adhered to the principles of the Helsinki Declaration. The studies involving human participants were reviewed and approved by the Institutional Review Board at the Maastricht University Medical Centre+ (NL70787.068.19). All patients were 18 years or older and provided written informed consent.

### Study procedures

Patient characteristics were collected, and clinical routine tests were performed before ablation, among them physical examination, a 10-second 12-channel electrocardiogram (ECG), and a computed tomography (CT) scan. In addition, several research-related procedures were performed on top of standard clinical care for detailed phenotyping of the patients. These included blood draw at the day of enrollment, as well as preprocedural intermittent ECG monitoring. Patients were ablated between 3 and 12 weeks after enrollment, and rhythm monitoring was performed for at least 12 months after ablation.

This study reports results of a cross-sectional analysis of the baseline data, with focus on blood biomarkers and preprocedural AF burden.

### Preprocedural AF burden

For preprocedural rhythm monitoring, patients were instructed to record single-lead handheld ECGs 3 times daily before ablation for a total duration of 4 weeks. In case of onset or relief of symptoms, patients were instructed to make additional recordings. ECGs were collected using the MyDiagnostick device (MyDiagnostick 1001R, Applied Biomedical Systems, Maastricht, The Netherlands). For this analysis, we included patients who had at least 14 days of ECG recordings. AF burden was calculated by dividing the number of single-lead ECGs recorded in AF by the total number of single-lead ECGs. Because of different definitions of AF burden in previous literature, an additional definition of AF burden was calculated—dividing the number of days in AF through the total number of days measured, based on a recent review of Becher et al.^17^

### Biomarker levels

Blood samples were collected in ethylenediaminetetraacetic acid tubes and stored at the local Biobank after centrifugation at 2000*g* for 10 minutes at 20 °C. Plasma was kept at –80 °C until analysis.

The biomarker panel was determined based on a Cochrane search in combination with a Delphi questionnaire answered by AF experts participating in the European CATCH ME consortium.^8^ The panel consisted of BMP10, Ang-2, FGF23, Dickkopf-related protein 3 (DKK3), Endocan endothelial cell-specific molecule 1 (ESM-1), insulin-like growth factor-binding protein 7 (IGFBP-7), B-type natriuretic peptides (NT-proBNP, total NT-proBNP), hs-TNT, growth differentiation factor 15 (GDF-15), interleukin 6 (IL-6), fatty acid–binding protein 3 (FABP3), Myosin binding protein C3 (MyBPC3), and cancer antigen-125 (CA-125) (Table 1).

**Table 1:** The different biomarkers and their possible working mechanism

Biomarker	Full name of biomarker	Pathophysiological mechanism
BMP10	Bone morphogenetic protein 10	Fibrosis
Ang-2	Angiopoietin-2	Endothelial dysfunction
FGF23	Fibroblast growth factor 23	Fibrosis
DKK3	Dickkopf-related protein 3	Kidney function
ESM-1	Endothelial cell specific molecule 1	Endothelial dysfunction
IGFBP-7	Insulin-like growth factor-binding protein 7	Senescence, vascular homeostatis, inflammation
NT-proBNP	Probain natriuretic peptide	Myocardial stretch
Total NT-proBNP	N-terminal fragment of probrain natriuretic peptide	Myocardial stretch
hs-TNT	High-sensitive troponin T	Myocardial damage
GDF-15	Growth/differentiation factor 15	Myocardial injury, hypoxia
IL-6	Interleukin-6	Inflammation
FABP3	Fatty acid binding protein 3	Myocardial injury
MyBPC3	Myosin-binding protein C3	Myocardial injury
CA-125	Cancer antigen 125	Inflammation

Biomarkers were measured using immunoassays developed by Roche Diagnostics. Cobas Elecsys® was used for NT-proBNP, hs-TNT, IL-6, GDF-15, and CA-125. Pre-commercial Elecsys® immunoassays were used for quantifying BMP10, Ang-2, ESM-1, FABP3,MyBPC3, FGF23, IGFBP-7, and total NT-proBNP. The biomarker quantification took place at Roche Diagnostics, Penzberg, Germany.^3^

### Statistical methods

We visualized the data to test for correlations through a correlation heatmap. The distribution of continuous data was visualized by histograms and qq-plots. Baseline characteristics are presented with means and their standard deviations or median (interquartile range [IQR]). Associations between biomarker levels and clinical characteristics were analyzed using linear regression models. For multivariable models, we performed elastic net linear regression analysis (R statistics; package glmnet, version 8) for each biomarker, with the biomarker levels as the dependent variable and the clinical determinants as independent variables.^18^ The following clinical characteristics (n = 64) were used as data source: demographic characteristics, comorbidities and risk factors, AF symptoms and triggers, physical examination measurements (blood pressure and anthropometric measurements), standard electrocardiogram parameters, and estimated glomerular filtration rate/creatinine (Table 2). AF burden was calculated by dividing the number of single-lead ECGs recorded in AF through the total number of single-lead ECGs. A Spearman rank correlation test was performed to assess the monotonic association between AF burden based on the number of recordings and AF burden in terms of percentage in days with AF.

**Table 2:** Baseline characteristics and biomarker values

	Overall
Total population	508
Female sex (%)	158 (31.1)
Age (mean [SD])	63.4 (±9.1)
Use of antiarrhythmic medication (%)	327 (64.4)
Medical history	
Heart failure (%)	77 (15.2)
Hypertension (%)	225 (44.3)
Previous ischemic stroke (%)	21 (4.1)
Hemorrhagic stroke (%)	2 (0.4)
Transient ischemic attack (%)	30 (5.9)
Peripheral vascular embolism (%)	9 (1.8)
Peripheral artery disease (%)	8 (1.6)
Coronary artery disease (%)	82 (16.1)
Myocardial infarction (%)	18 (3.5)
Diabetes Mellitus (%)	41 (8.0)
Hypercholesterolemia (%)	115 (22.6)
Family history of AF (%)	139 (27.4)
Chronic obstructive pulmonary disease (%)	29 (5.7)
Decreased kidney function (%)	20 (4.0)
Thyroid dysfunction (%)	38 (7.5)
Sleep apnea syndrome (%)	94 (18.5)
Active smoker (%)	64 (12.6)
Moderate or severe mitral regurgitation	13 (2.6)
Normal glomerular filtration rate (eGFR > 90 mL/min/m^2^)	115 (22.6)
Baseline (anthropometric) measurements	
Length (mean [SD])	1.78 (0.10)
Weight (mean [SD])	89.2 (15.1)
Body mass index (mean [SD])	28.2 (4.1)
Body surface area (mean [SD])	2.1 (0.20)
Fat percentage (mean [SD])	30.3 (8.6)
Muscle percentage (mean [SD])	30.3 (4.4)
Visceral fat (mean [SD])	11.9 (4.1)
Rest metabolism (mean [SD])	1747.5 (±210.9)
Systolic blood pressure (mean [SD])	138.7 (±18.1)
Diastolic blood pressure (mean [SD])	80.6 (10.6)
Atrial fibrillation history	
Persistent atrial fibrillation (%)	154 (30.3)
CHADS2VASc (median [IQR])	2 [1–3]
Previous ablation the left atrium (%)	54 (10.6)
Atrial fibrillation history: Triggers	
Alcohol intake (%)	50 (9.8)
Infection (%)	12 (2.4)
Exercise or rest after exercise (%)	88 (17.3)
Coffee intake (%)	17 (3.4)
Emotions or stress (%)	82 (16.1)
Large meal (%)	13 (2.6)
Sleep (%)	57 (11.2)
Atrial fibrillation history: symptoms	
Palpitations (%)	346 (68.1)
Dyspnea (%)	157 (31.0)
Chest pain or discomfort (%)	96 (18.9)
Restless feeling (%)	83 (16.3)
Fatigue (%)	129 (25.4)
Dizziness or lightheadedness (%)	129 (25.4)
Syncope (%)	18 (3.5)
Polyuria (%)	11 (2.2)
Baseline electrocardiogram	
Atrial fibrillation (AF) rhythm on ECG (%)	126 (24.8)
Heartrate (mean [SD])	67.3 (±15.6)
PQ (mean [SD]), ms	169.9 (±24.7)
QRS (mean [SD]), ms	97.2 (±11.85)
QTc (mean [SD]), ms	432.51 (±30.61)
Biomarker values	
BMP10 (mean [SD]), ng/mL	2.03 (±0.45)
Ang-2 (mean [SD]), ng/mL	2.34 (±1.63)
FGF23 (mean [SD]), pg/mL	186.17 (±149.74)
DKK3 (mean [SD]), pg/mL	52.69 (±12.15)
ESM-1 (mean [SD]), pg/mL	2070.94 (±698.30)
IGFBP-7 (mean [SD]), ng/mL	90.80 (±17.68)
NT-proBNP (mean [SD]), pg/mL	352.13 (±481.98)
Hs-TNT (mean [SD]), pg/mL	9.13 (±5.43)
GDF-15 (mean [SD]), pg/mL	1190.57 (±853.30)
IL-6 (mean [SD]), pg/mL	3.94 (±11.76)
MyBPC3 (mean [SD]), pg/mL	14.43 (±60.33)
FABP3 (mean [SD]), ng/mL	24.64 (±8.13)
CA125 (mean [SD]), U/mL	13.21 (±8.44)
Total NT-proBNP (mean [SD]), pg/mL	1399.95 (±1335.63)

Ang-2 = angiopoietin-2; BMP10 = bone morphogenetic protein 10; DKK3 = Dickkopf-related protein 3; ECG = electrocardiogram; ESM-1 = Endocan endothelial cell-specific molecule 1; FABP3 = fatty acid–binding protein 3; FGF23 = fibroblast growth factor 23; GDF-15 = growth differentiation factor 15; hs-TNT = high sensitive Troponin-T; IGFBP-7 = insulin-like growth factor-binding protein 7; IL-6 = interleukin-6; MyBPC3 = Myosin binding protein C3; NT-proBNP = N-terminal pro-brain natriuretic peptide; SD = standard deviation.

The biomarker values were first standardized to be able to compare coefficients between the different models and biomarkers. Outliers in biomarker levels were removed and replaced by imputed values based on linear regression models for imputation of continuous variables and logistic regression for categorical and Boolean variables.

The model selected the most important clinical determinants per biomarker and created multivariable linear regression models. The correlation coefficients of the associations of clinical features with biomarkers levels were plotted in heatmaps. In addition, we plotted histograms for the partial *R*^*2*^ values associated with each variable per biomarker to depict the extent to which each variable contributed to explain the overall variance of the biomarker values in the conditional model. A 2-sided *P* < .05 was considered significant in all analyses.

## Results

### Baseline characteristics

During the study period, 508 patients met the inclusion and exclusion criteria, were included in the cohort, and had blood samples available from the baseline visit. Their baseline characteristics are presented in Table 2. Mean age was 63 ± 9 years, and 31.1% patients were female. In 354 patients (69.7%), paroxysmal AF had been reported, and 154 patients (30.3%) had persistent AF. In 140 (27.6%) patients, AF rhythm was observed during blood draw (Table 2). The mean values of the biomarkers of all the patients are shown in Table 2. Heart failure was reported in 77 patients (15.2%). In a subanalysis of 413 patients in whom echocardiographic left ventricular ejection fraction (LVEF) was available, 55 patients (13%) had an LVEF below 50%, and 3 (<1%) patients had LVEF below 30%.

### Biomarker associations with baseline characteristics and with AF rhythm during blood draw

**Figure 1:**
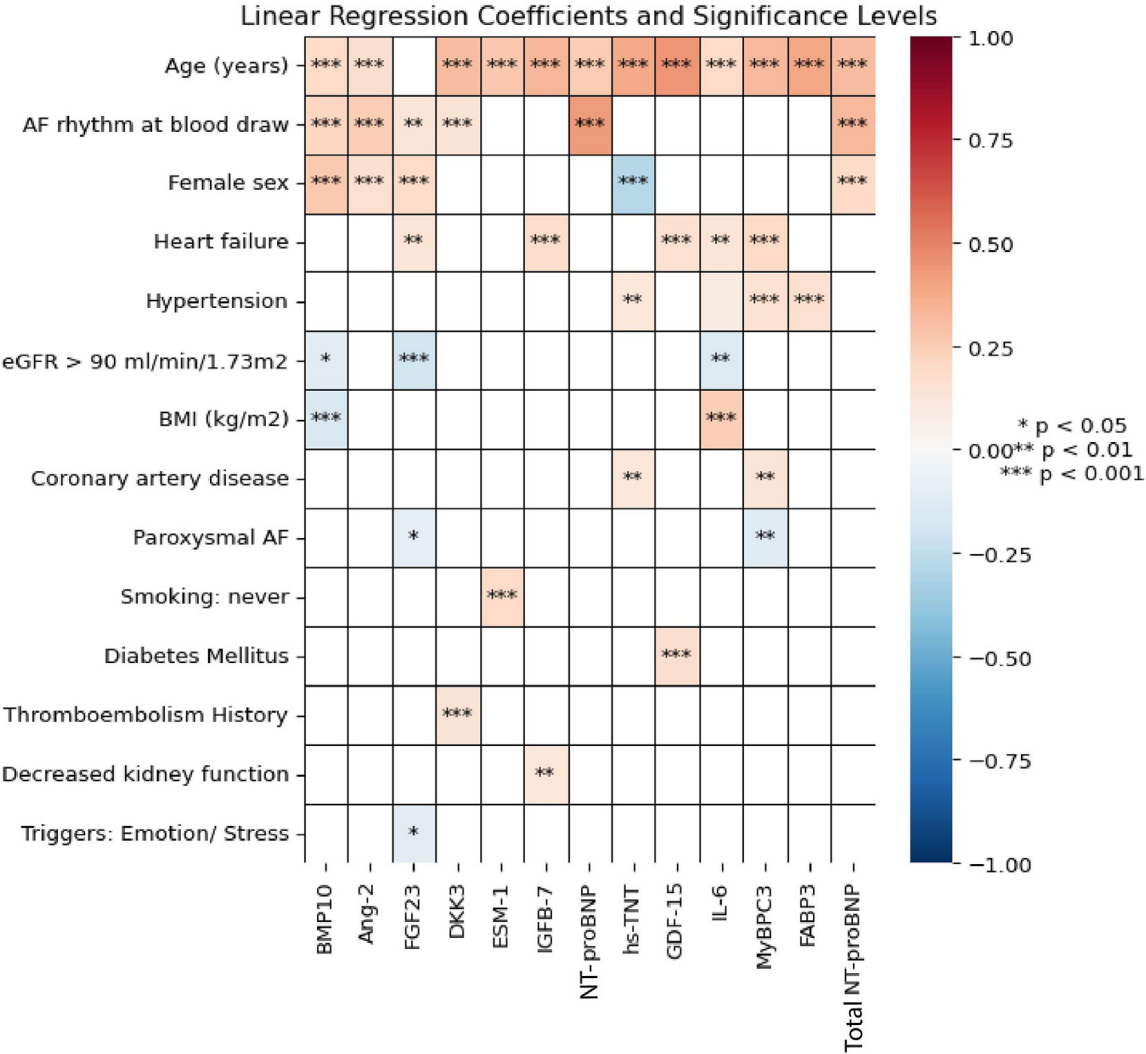
Standardized biomarker values and their associations with baseline characteristics with rhythm at blood draw. Linear regression coefficients and significance levels of associations between standardized biomarkers (columns) and baseline characteristics (rows) in participants with including rhythm at the time of blood draw. *Red* represents positive correlations and *blue* represents negative correlations. The color intensity indicates the strength of the associations. Significance levels: ∗*P* < .05; ∗∗*P* < .01; ∗∗∗*P* < .001.

**Figure 2:**
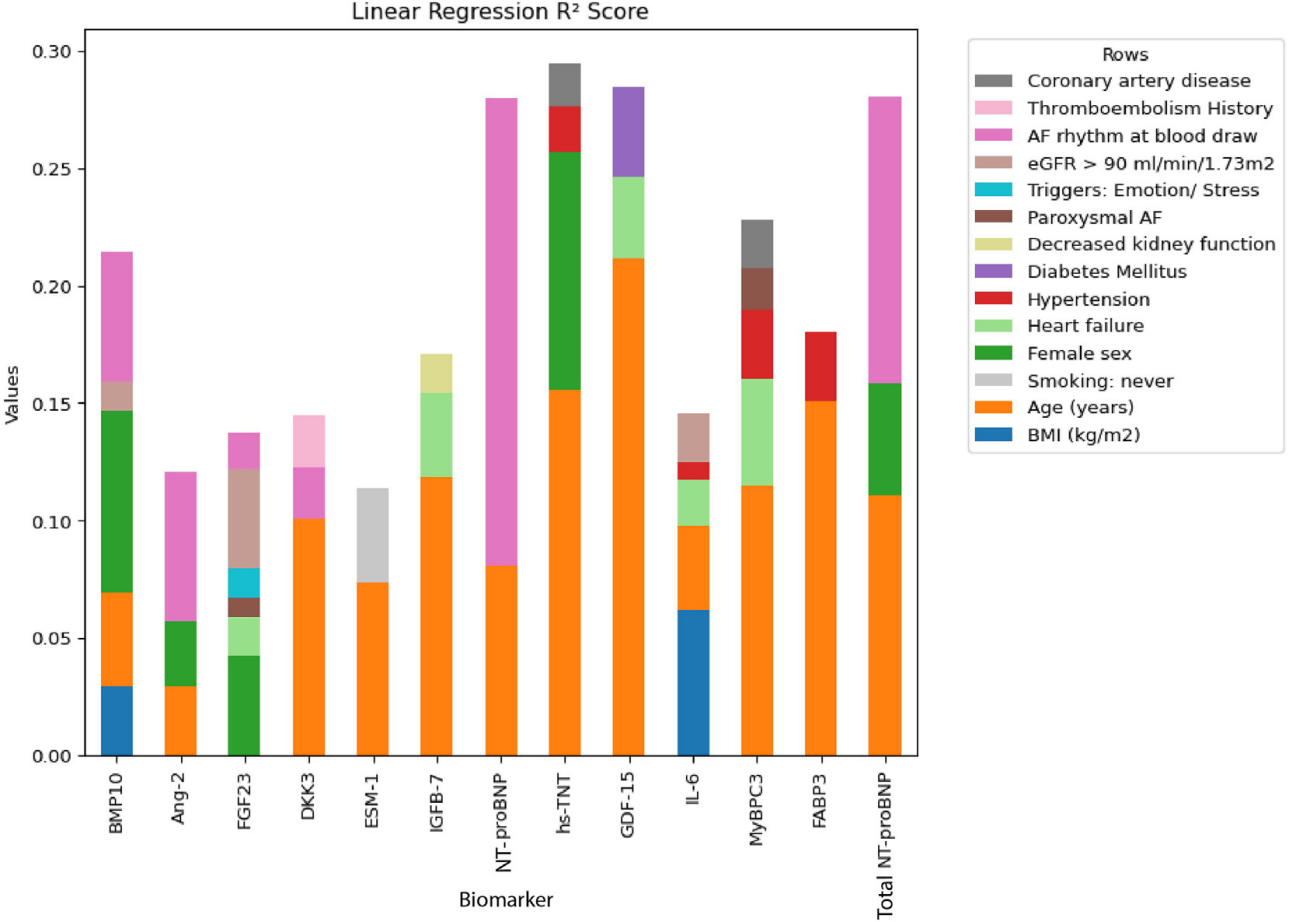
Standardized biomarker values and the linear regression partial *R*^*2*^ scores with rhythm at blood draw. Linear regression partial *R*^*2*^ scores for standardized biomarkers (*columns*) in participants with rhythm at the time of blood draw included. Each *bar* represents the contribution of different baseline characteristics (color-coded) to the variance explained in each biomarker's value, demonstrating their relative influence on each biomarker’s *R*^*2*^ score.

The coefficients of the clinical parameters per biomarker including AF rhythm during blood draw are shown in Figure 1. Age was associated with higher biomarker levels of BMP10, DKK3, ESM-1, IGFB7, NT-proBNP, hs-TNT, and GDF15. AF rhythm during blood draw was associated with higher biomarker levels of BMP10, Ang-2, FGF23, DKK3, NT-proBNP, and total NT-proBNP. Female sex was associated with higher biomarkers levels of BMP10, Ang-2, FGF23, and total NT-proBNP and negatively associated with Hs-TNT. Heart failure was associated with FGF23, IGFB-7, GDF-15, IL-6, and MyBPC3 (Figure 1). In Figure 2, the partial *R*^*2*^s are visualized from the clinical determinants models, taking rhythm during blood draw into consideration. In Figure 2, age is the variable that contributes most to explain the overall variance of the following biomarker levels in the conditional model: DKK3, ESM-1, IGFBP-7, hs-TNT, GDF-15, MyBPC3, FABP3, and NT-proBNP. Female sex contributes the most to the levels of BMP-10 and FGF23. After age, female sex contributed most to the biomarker values of hs-TNT. Furthermore, AF rhythm at blood draw contributed most to the levels of total NT-proBNP, NT-proBNP, and Ang-2. After female sex, AF rhythm at blood draw was the trait most strongly associated with the levels of BMP-10. After age, AF rhythm at blood draw was strongly associated with levels of total NT-proBNP. Notably, body mass index (BMI contributed most to the level of IL-6.

### Biomarker values and their associations with baseline characteristics without AF rhythm during blood draw

In Supplementary Figure A1, the clinical determinants associated with biomarker levels are shown without including AF rhythm during blood draw. Notably, age was still the strongest clinical determinant of higher levels of BMP10, Ang-2, DKK3, ESM-1, IGFB-7, NT-proBNP, Hs-TNT, GDF-15, IL-6, MyBPC3, FABP3, and total NT-proBNP. In Supplementary Figure A2, age is the variable that contributes most to explain the overall variance of the following biomarkers in the conditional model: Ang-2, DKK3, ESM-1, IGFBP-7, NT-proBNP, hs-TNT, GDF15, MyBPC3, FABP3, and total NT-proBNP. When comparing Figure 2 and Supplementary Figure A2, it becomes evident that the exclusion of AF rhythm during blood draw did not affect the observed association of biomarkers with clinical features. This finding underscores the robustness of the associations between biomarkers and clinical determinants.

### Biomarker associations with baseline characteristics, including preprocedural AF burden

In 389 patients, information on AF burden was available. Their baseline characteristics were similar to those of the main cohort. Their mean age was 64 ± 9 years (*P* = .91), and 33.1% (*P* = .07) of the patients were female. In 273 patients (70.2%) paroxysmal AF had been reported, and 116 (29.8%) patients had persistent AF (*P* = .65). Heart failure was reported in 58 patients (14.9 %) (*P* = .77). In 102 (26.2%) (*P* = .22) patients, AF rhythm was observed during blood draw.

**Figure 3:**
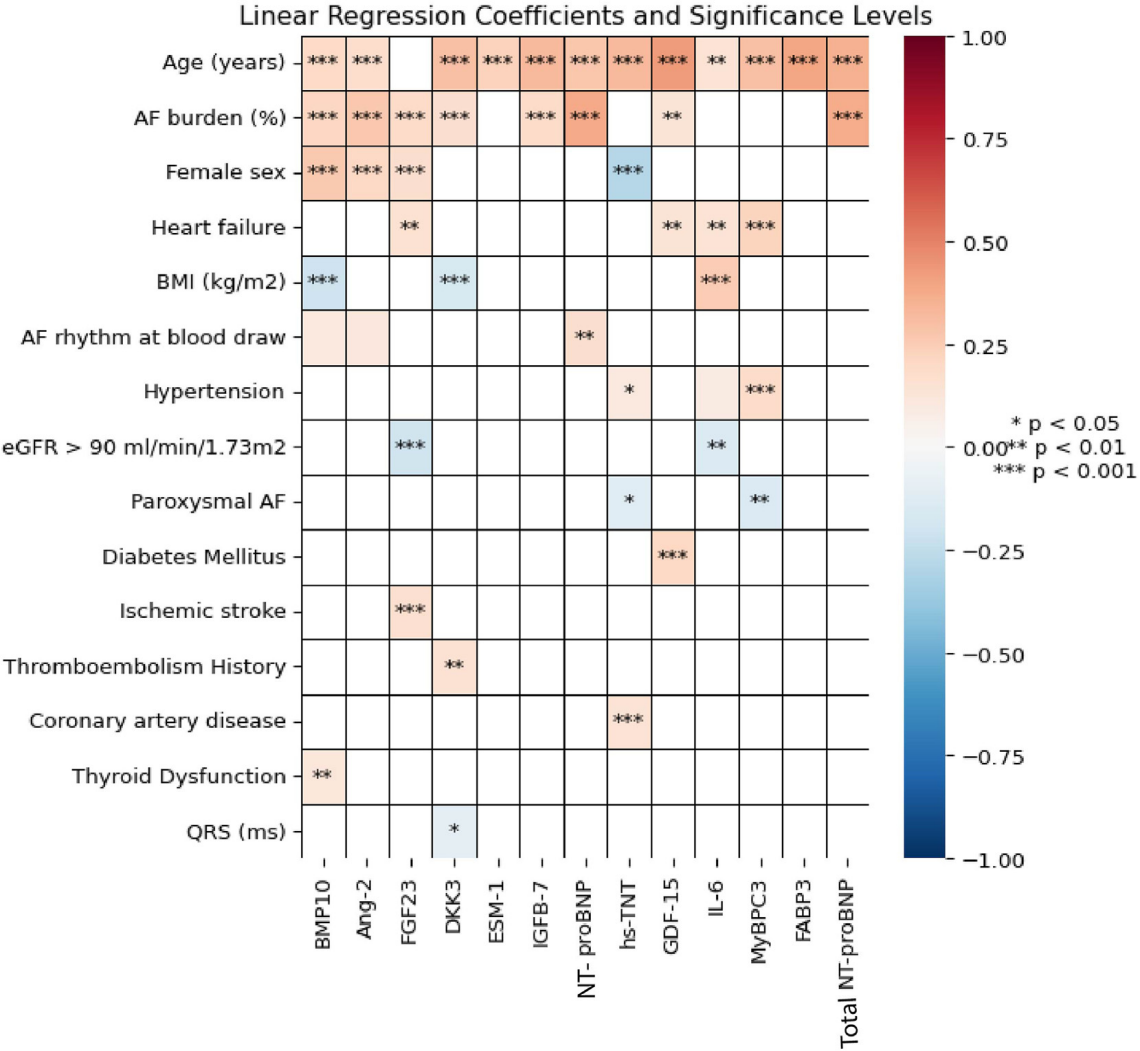
Standardized biomarker values and their associations with baseline characteristics with preprocedural AF burden. Linear regression coefficients and significance levels of associations between standardized biomarkers (*columns*) and baseline characteristics (*rows*) in participants with AF burden. Red represents positive correlations and blue represents negative correlations. The color intensity indicates the strength of the associations. Significance levels: ∗*P* < .05; ∗∗*P* < .01; ∗∗∗*P* < .001.

**Figure 4:**
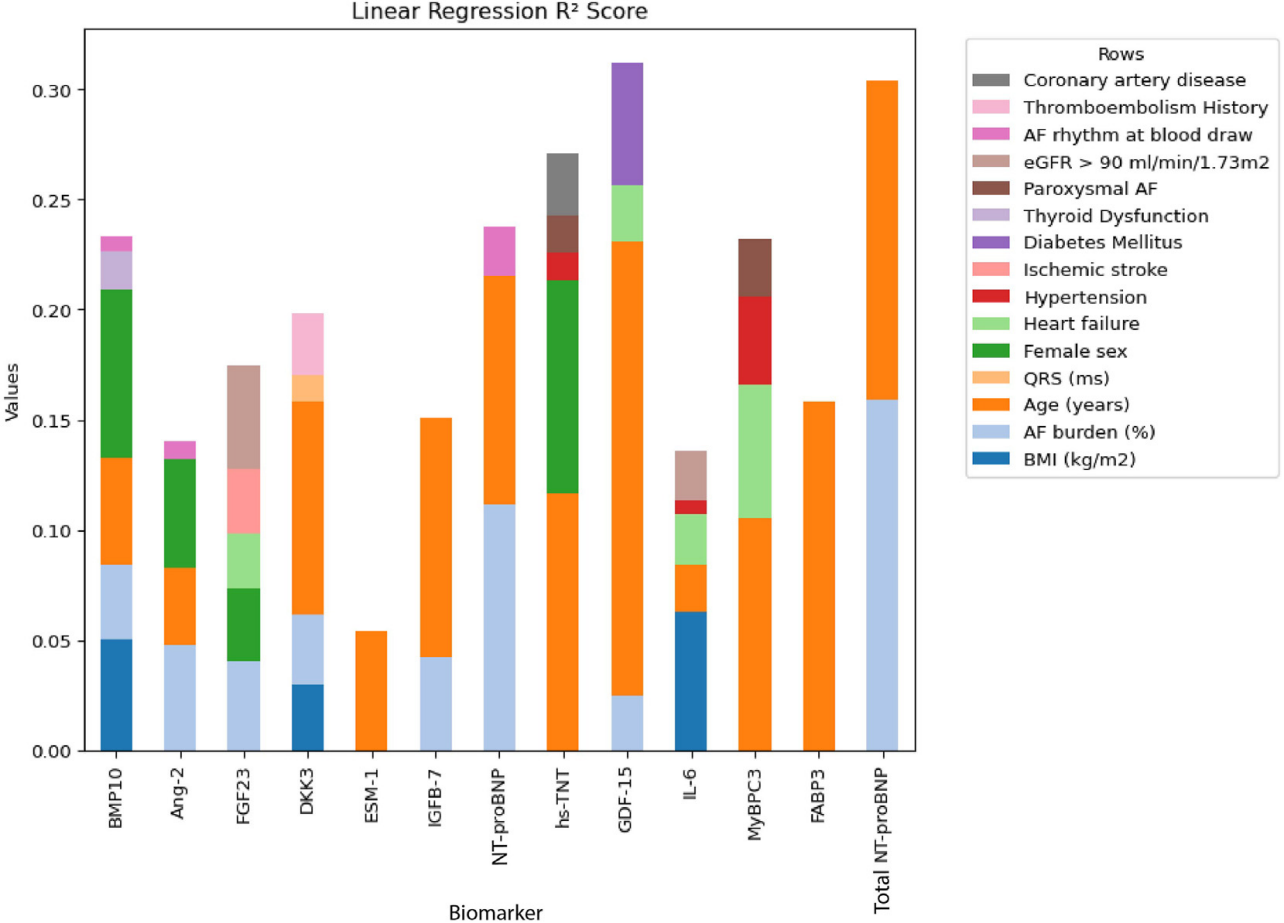
Standardized biomarker values and the linear regression partial *R*^*2*^ scores with pre-procedural AF burden. Linear regression partial *R*^*2*^ scores for standardized biomarkers (*columns*) in participants with AF burden. Each bar represents the contribution of different baseline characteristics (color-coded) to the variance explained in each biomarker’s value, demonstrating their relative influence on each biomarker’s *R*^*2*^ score.

The clinical determinants (including AF burden) associated with the various biomarker levels are shown in Figure 3. In this subanalysis, age was still the strongest clinical determinant of higher biomarker levels of BMP10, Ang-2, DKK3, ESM-1, IGFBP-7, NT-proBNP, Hs-TNT, GDF-15, IL-6, MyBPC3, FABP3, and total NT-proBNP. Similar to in the overall cohort, female sex was associated with higher levels of BMP10, Ang-2, and FGF23 and lower levels of hs-TNT. Notably, AF burden was strongly associated with the biomarker levels of BMP10, ANG2, FGF23, DKK3, IGFBP-7, NT-proBNP, GDF-15, and total NT-proBNP. As shown in Figure 4, age was the variable that contributed most to explain the overall variance of the following biomarker levels in the conditional model: DKK3, ESM-1, IGFBP-7, GDF-15, MyBPC3, and FABP3. Sex contributed most to the levels of BMP-10 and hs-TNT. After age, sex was clinical trait most strongly associated with hs-TNT. AF burden contributed most to the levels of NT-proBNP, total NT-proBNP, and FGF23.

When using the definition of AF burden based on number of days in AF, we found that within our cohort the AF burden based on the number of recordings and AF burden in terms of percentage in days were strongly correlated (ρ = 0.99, *P* < .001). Age was again the variable that contributed most to explain the overall variance of the biomarker levels. AF burden in terms of percentage in days was most strongly associated with the biomarker levels of BMP10, ANG2, FGF23, DKK3, IGFB-7, NT-proBNP, and total NT-proBNP. AF burden in terms of percentage in days contributed the most to the levels of FGF-23, NT-proBNP, and total NT-proBNP.

To assess whether AF duration has an effect on biomarker levels in patients with persistent AF, we performed a subanalysis. Among the 154 patients with persistent AF, AF duration—defined as the time between first AF diagnosis and the baseline visit—was available for 153 patients. After adjusting for sex and age, we did not find a significant association between biomarker levels and AF duration in patients with persistent AF.

## Discussion

In this study, we studied the association of a recently developed panel of biomarkers with the clinical profile of patients undergoing ablation for AF recruited in the ISOLATION study.

Our main findings are:

1 AF burden before AF ablation was strongly associated with higher biomarker levels of BMP-10, ANG-2, FGF23, DKK3, IGFB-7, NT-proBNP, GDF-15, and total NT-proBNP.
2 Biomarker levels of BMP10, Ang-2, FGF23, DKK3, pro-BNP, and total NT-proBNP were associated with AF present during blood draw.
3 Overall, age was the dominant clinical feature associated with circulating biomarker levels.
4. Biomarkers associated with (atrial) fibrosis were associated with female sex, and the myocardial damage marker hs-TNT is associated with male sex.

### AF burden and AF during blood draw

The association between preprocedural AF burden and biomarker levels had not been investigated thoroughly. Our findings suggest a clear association between preprocedural AF burden and elevated levels of biomarkers. This might reflect more cardiac stress in patients with higher AF burden. In a subanalysis of CASTLE-AF, AF burden at 6 months was a predictor of hard clinical outcomes in patients with heart failure undergoing ablation. The authors speculate that a higher AF burden reflects advanced atrial remodeling,^19^^,^^20^ which may be reflected in biomarker levels. Our findings are also in line with a previous analysis in a relatively small subset of 27 patients from the CRYPTO-AF study.^10^ In that study, AF burden was derived from Holter monitoring and associated with higher levels of NT-ProBNP and FGF23.^10^ Our study shows that, in principle, biomarkers might be used to estimate AF burden and therefore could have a role in guiding anticoagulation therapy in the future. It appears very worthwhile to study the accuracy of AF burden estimation using biomarkers, possibly in combination with other clinical traits, such as ECG markers.

Notably, AF rhythm during blood draw emerged as a determinant of plasma levels of BMP10, Ang-2, FGF23, DKK3, NT-proBNP, and total NT-proBNP. Thus, when using those biomarker levels for individual risk assessment of biomarker levels, the rhythm during blood draw should be included in the multivariable model. However, the overall pattern of associations between biomarkers and clinical features was not affected by the rhythm during blood draw. The latter observation suggests that the pathophysiological mechanisms present in patients undergoing AF ablation identified by biomarkers are not dependent on the rhythm during blood draw.

### Age

In the ISOLATION cohort, we found an independent association between age and most of the biomarkers (including BMP10, Ang-2, DKK3, ESM1, IGFBP7, pro BNP, hs-TNT, GDF15, IL-6, MyBP3, FABP3, total NT-proBNP). These associations remained significant after correcting for co-morbidities that are associated with AF. Because biomarkers represent the presence of certain pathophysiological mechanisms, our data suggest that age in itself is associated with multiple pathophysiologic mechanisms such as inflammation, endothelial dysfunction, myocardial injury, and atrial fibrosis. For example, total NT-proBNP and NT-proBNP, which are associated with atrial stretch and dilatation, have been shown to increase with ageing.^10^^,^^21^^,^^22^ Although the contribution of each single pathophysiological mechanisms to the increased AF propensity might be limited, the multitude of associations between age and biomarkers supports the view that many and different pathophysiological mechanisms synergistically enhance inducibility and perpetuation of AF, causing altogether the strong association between age and prevalence of AF.^13^

### Atrial fibrosis

One of the biomarkers investigated, BMP10 is a protein expressed by right atrial myocytes and suppressed by the PITX2 gene.^23^ In a large cohort study with more than 15,000 patients of Hijazi et al,^5^ BMP10 has recently been linked to ischemic stroke in patients with AF and recurrence after AF ablation. Although animal studies have shown that BMP10 might have a protective function in mice with heart failure,^24^ the precise working mechanism of BMP10 remains unclear. Within our cohort, we find a strong association of low BMI with BMP10 levels. These findings are in line with the large cohort study of 15,000 patients.^5^ A recent study from the CATCH-ME consortium has shown that endomysial fibrosis was associated with female sex, heart failure, and history of AF.^25^ Furthermore, a recent study across a heterogeneous cohort of patients undergoing cardiac surgery has shown that elevated BMP10 levels correlate with a history of persistent AF, incidence of late postoperative AF, and the presence of endomysial fibrosis in the left atrial tissue samples.^6^

In addition to BMP10, FGF23 reflects in atrial fibrosis.^14^ Both, BMP10 and FGF23 were consistently higher in female patients, indicating a potential role of atrial fibrosis in women with AF. The results align with the findings of the CATCH-ME consortium, indicating that fibrosis in human atria is more pronounced in women than in men with AF.^25^

### Myocardial injury

Hs-TNT, GDF 15,^10^ and IGFBP-7 are associated with myocardial injury, the latter particularly in patients with heart failure.^26^ In our cohort, hs-TNT levels were elevated in men, suggesting a higher prevalence or extent of myocardial damage in men than in women. Conversely, MyBPC3, another myocardial injury marker, is associated with heart failure, persistent AF, and hypertension but not with sex. The association with distinctly different clinical traits of these injury markers raises questions regarding the nuanced pathophysiological pathways underlying myocardial damage in our patient population. In the study of Røsjø et al,^27^ in which cardiac troponin concentrations were assessed in women and men with AF, troponin levels in men with AF were also elevated compared with those of women in AF.^27^

### Endothelial dysfunction

Ang-2 and ESM-1 are novel biomarkers of endothelial inflammation and vascular remodeling in patients with AF.^28^ Studies strongly suggest a potential role of endothelial and vascular dysfunction in the genesis of AF or a bidirectional association between these diseases.^29^^,^^30^ From a mechanistic point of view, the association between AF and endothelial dysfunction involves enhanced oxidative stress, myocardial ischemia, and increased vascular shear stress, collectively contributing to the pathophysiology of AF.^31^ Our study demonstrates an association of Ang-2 with AF burden, also supporting the contribution of endothelial dysfunction to AF occurrence.

### BMI and inflammation

IL-6, CA-125, and ESM-1 are biomarkers associated with inflammation. Adipose tissue is a known source of inflammatory mediators that attract inflammatory cells and activate fibroblasts.^32^ Furthermore, adipose tissue also may directly impede atrial conduction.^33^ In our cohort, IL-6 was associated with higher BMI, consistent with the role of inflammation caused by inflammatory cytokines released from adipose tissue.

CA-125 is conventionally known as a tumor marker. Although studies have indicated elevated CA-125 levels in patients with AF compared with those in sinus rhythm, uncertainty remains in the relationship between CA-125 and AF.^34^ In our study population, we did not observe any association of the clinical determinants with CA-125 levels; therefore, these data were not included in the heatmaps.

### Heart failure

We were surprised that we did not find an association between heart failure and total NT-proBNP or NT-proBNP. However, in the subanalysis of 413 patients in whom LVEF was available, only 58 patients (13%) had an LVEF below 50%, indicating that the power of our study to detect associations between biomarkers and heart failure was limited. Furthermore, NT-proBNP levels in patients with AF might already be elevated, which could lead to finding no differences in patients with and without HF. In patients with AF, the diagnostic value of NT-proBNP for HF without reduced rejection fraction is very limited.^35^

### Strengths and limitations

We have a prospective and well-characterized cohort. The observational cohort study might include casual inference. Furthermore, there is no clear definition of AF burden in the literature. We choose a number of recordings in AF. Within our dataset, we did an additional analysis and investigated a different definition of AF burden based on a recent published review of Becher et al.^17^ Within this review, the suggestion is to calculate AF burden based on number of days in AF divided by the total number of days measured. Within our cohort, there was a very strong positive correlation between AF burden from recordings and days in AF (ρ = 0.99, *P* < .001), with similar outcomes. The results were not hardly influenced by the way the AF burden was quantified. Further investigation for a clear definition of AF burden is needed.

### Clinical impact and conclusions

Our study provides valuable insights into the relationships between clinical determinants and biomarker levels in patients undergoing AF ablation. We observed independent associations between biomarkers with preprocedural AF burden, age, sex, and AF rhythm during blood draw. In women, profibrotic biomarkers were frequently higher, whereas male patients exhibited markers indicative signs of myocardial injury. AF burden is a very strong determinant of many biomarkers, stressing the importance of preprocedural AF burden as a routine clinical determinant in future biomarker studies.
