# Supplementary material for: Association of atrial fibrillation burden and clinical profile with blood biomarkers: Results from the ISOLATION Ablation Cohort
Source: Heart Rhythm O2. 2025 Feb 28;6(5):661–70. doi: 10.1016/j.hroo.2025.02.017 (PMC12147586; doi:10.1016/j.hroo.2025.02.017)
Supplement: Supplementary Figures [file mmc1.docx]

- **Appendices**
- **Figure A1. Standardized biomarker values and their associations with baseline characteristics without rhythm at blood draw**.
- **Figure A2. Standardized biomarker values and the linear regression partial R^2^ scores without rhythm at blood draw**

**
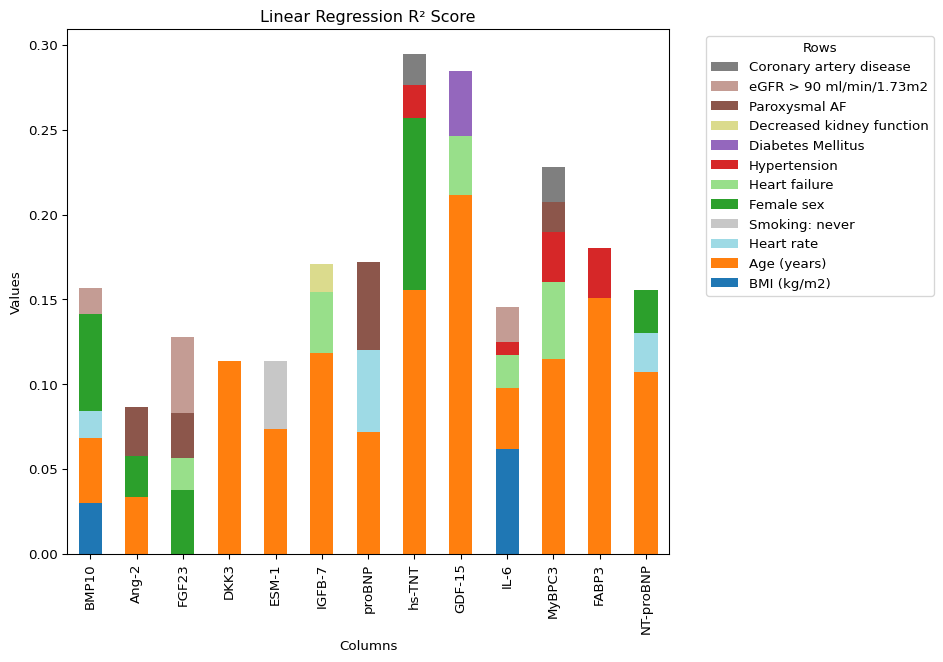
Figure A1. Figure**
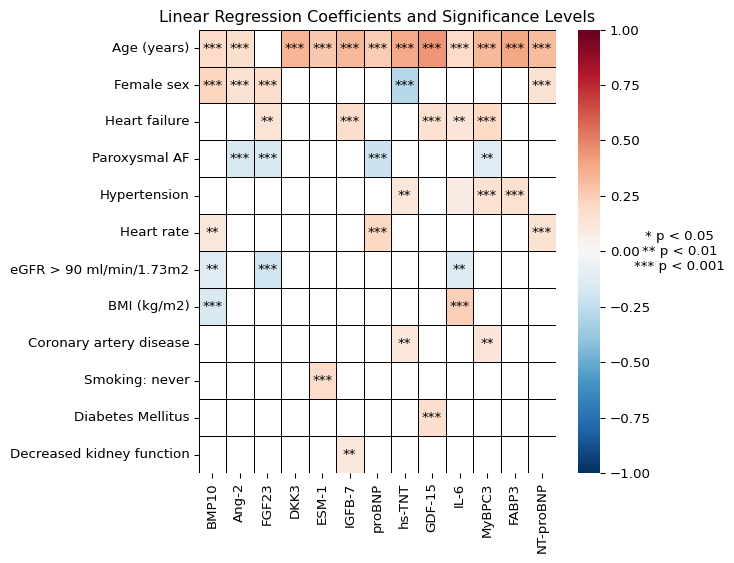
**A2.**

NT-

**Figure A2. Standardized biomarker values and the linear regression partial R^2^ scores without rhythm at blood draw** *Legend: Linear regression partial R² scores for standardized biomarkers (columns) in participants without rhythm at the time of blood draw included. Each bar represents the contribution of different baseline characteristics (color-coded) to the variance explained in each biomarker's value, demonstrating their relative influence on each biomarker's R² score.*

NT-

Total

Total

**Figure A1. Standardized biomarker values and their associations with baseline characteristics without rhythm at blood draw**. *Legend: Linear regression coefficients and significance levels of associations between standardized biomarkers (columns) and baseline characteristics (rows) in participants without including rhythm at the time of blood draw. Red represents positive correlations and blue represents negative correlations. The color intensity indicates the strength of the associations. Significance levels: *p < 0.05, **p < 0.01, ***p < 0.001.*
